# Dendrimeric Antigens for Drug Allergy Diagnosis: A New Approach for Basophil Activation Tests

**DOI:** 10.3390/molecules23050997

**Published:** 2018-04-24

**Authors:** Noemi Molina, Angela Martin-Serrano, Tahia D. Fernandez, Amene Tesfaye, Francisco Najera, María J. Torres, Cristobalina Mayorga, Yolanda Vida, Maria I. Montañez, Ezequiel Perez-Inestrosa

**Affiliations:** 1 Departamento de Química Orgánica, Universidad de Málaga—IBIMA, 29071 Málaga, Spain; nmolina@uma.es (N.M.); najera@uma.es (F.N.); 2 Andalusian Centre for Nanomedicine and Biotechnology-BIONAND, Parque Tecnológico de Andalucía, 29590 Málaga, Spain; amso_almaden@hotmail.com (A.M.-S.); tahia.fernandez@ibima.eu (T.D.F.); amenet2009@gmail.com (A.T.); mjtorresj@ibima.eu (M.J.T.); mayorga.lina@gmail.com (C.M.); 3 Research Laboratory, IBIMA—Regional University Hospital of Málaga—UMA, 29009 Málaga, Spain; 4 Allergy Unit, IBIMA—Regional University Hospital of Málaga—UMA, 29009 Málaga, Spain

**Keywords:** PAMAM, dendrimeric antigens, penicillin, drug allergy, basophil activation tests

## Abstract

Dendrimeric Antigens (DeAns) consist of dendrimers decorated with multiple units of drug antigenic determinants. These conjugates have been shown to be a powerful tool for diagnosing penicillin allergy using in vitro immunoassays, in which they are recognized by specific IgE from allergic patients. Here we propose a new diagnostic approach using DeAns in cellular tests, in which recognition occurs through IgE bound to the basophil surface. Both IgE molecular recognition and subsequent cell activation may be influenced by the tridimensional architecture and size of the immunogens. Structural features of benzylpenicilloyl-DeAn and amoxicilloyl-DeAn (G2 and G4 PAMAM) were studied by diffusion Nuclear Magnetic Resonance (NMR) experiments and are discussed in relation to molecular dynamics simulation (MDS) observations. IgE recognition was clinically evaluated using the basophil activation test (BAT) for allergic patients and tolerant subjects. Diffusion NMR experiments, MDS and cellular studies provide evidence that the size of the DeAn, its antigen composition and tridimensional distribution play key roles in IgE-antigen recognition at the effector cell surface. These results indicate that the fourth generation DeAns induce a higher level of basophil activation in allergic patients. This approach can be considered as a potential complementary diagnostic method for evaluating penicillin allergy.

## 1. Introduction

Dendrimers are highly branched and regular macromolecules with well-defined structures that attract considerable interest due to their potential applications in many fields of science. The three-dimensional architecture of dendrimeric systems confers them various intrinsic features such as structural homogeneity, integrity, controlled composition and high-density multidentate homogeneous terminal groups, ready for conjugation. These characteristics, added to their stability and versatility, mean that dendrimers have been used for many applications, such as sensing, catalysis, molecular electronics and photonics [1]. Moreover, dendrimer and dendron nanostructures represent ideal scaffolds for numerous bioapplications and hold great promise for the future of nanomedicine [2,3]. One important application is related to the study of allergic drug reactions. 

Allergic drug reactions are one of the most important health problems nowadays. Betalactam (BL) allergy is self-reported by approximately 10% of adverse drug reaction sufferers. A high proportion of these cases are mediated by immunoglobulin E (IgE), leading to a range of symptoms, from simple skin involvement to anaphylactic shock [4,5]. However, a variety of factors make the study of these reactions difficult, such as a lack of knowledge of the actual drug derivatives involved, changes to the pattern of hapten recognition over time in selected populations, the possibility of cross-reactivity between related chemical structures and a general increase in adverse patient responses due to environmental and genetic factors [6,7,8].

In addition, BL allergy has a complex diagnosis which is still not correctly addressed. Many BLs with different chemical structures exist and each patient has a unique IgE response. An individual can be allergic to one BL only, or cross-reactive, being allergic to BLs with the same or similar side chains, or to multiple, potentially structurally diverse BLs from different families [9,10,11]. A complete diagnostic procedure includes a detailed clinical history, which can be unreliable, followed by in vivo tests, including skin tests, which can have low sensitivity, and drug provocation testing, which poses some patient-risk, especially for severe reactions [12]. 

In vitro testing represents a more rational alternative, as it can potentially identify the drug responsible, allowing the physician to find a safe alternative and reducing the need to perform drug provocation testing. However, it is necessary to confirm the sensitivity, specificity and negative and positive predictive values for these in vitro tests in order to enable their implementation in clinical practice. The most common in vitro tests are based on the detection of specific IgE, either in serum (immunoassays) or bound to receptors on the surface of effector cells (basophil activation tests) [13]. 

BLs are haptens that need to be bound covalently to a carrier protein to induce an immune response. This hapten-carrier conjugate is used in immunoassays to quantify drug specific IgE (sIgE) in serum. The immunoassay is the most widely used in vitro test for the diagnosis of drug allergy. However, it has certain disadvantages, such as low sensitivity and the limited availability of commercial kits for a small range of BLs. Among non-commercial ones, Radio Allergo Sorbent Test (RAST) is the most commonly employed in research laboratories.

The nature of the carrier molecule is important for the development of an in vitro test for the detection of drug sIgE antibodies, as the conjugate it forms can influence IgE recognition. Poly-l-Lysine is the most widely used carrier molecule for the RAST, due to its accessibility, multivalency and ease of chemical functionalization for different haptens [14,15]. However, its inherent polydispersity complicates its precise chemical characterization and affects the reproducibility of the formed conjugate. To avoid these handicaps and to produce dense and reproducible hapten-carrier conjugates we proposed the use of PAMAM dendrimers [16] as carrier molecules. Their potential for emulating the carrier protein in hapten-carrier conjugates for IgE recognition in BL allergy has been confirmed using dendrimeric antigens (DeAns). DeAns were synthetized by incorporating benzylpenicilloyl (BPO, the antigenic determinant of benzylpenicillin) or amoxicilloyl (AXO; the equivalent of amoxicillin) groups in the dendrimer periphery. Bi-epitope DeAns have also been designed, including both BPO and AXO on the same macromolecule, which enabled the detection of sIgE from selective and cross-reactive patients [17]. The coupling of DeAns to different solid supports (cellulose disks, zeolites and silica particles) have allowed the determination of sIgE to penicillins by RAST, and this has been shown to have potential for diagnosis [18,19,20,21,22,23].

Although the nanotechnological advances achieved in immunoassay design are very promising [24,25], RAST assays have certain limitations, such as dependencies on a radioactive isotope, specific facilities and trained personnel. Thus, other techniques avoiding radioactivity are preferred by many research groups. Consequently, the basophil activation test (BAT), another in vitro diagnostic technique, has received increasing attention for the diagnosis of drug allergy [12,13]. 

Like other functional assays, BAT tries to mimic in vivo IgE-mediated cell activation and mediator release. This test is useful for evaluating IgE-mediated reactions for a variety of injectable drugs since there is no need to use drug-carrier conjugates. BAT is also valuable for the identification of the drug responsible for a reaction. However, its sensitivity depends on the drug involved, with values of around 55% reported for BLs [26,27,28].

BAT is based on the determination of activation markers expressed on basophil surface after the interaction of the drug with sIgE [13]. However, the lack of knowledge of activation mechanisms has hampered a wider clinical application. BLs are not capable of activating basophils by themselves. They require conjugation to a carrier molecule, generally present in the blood, that is big enough to allow cross-linking of two sIgE bound to the basophil surface. However, no information about the size and compositions of these conjugates is available. The use of well-defined hapten-carrier conjugates would be a valuable tool for the investigation of the mechanism through which the activation occurs. 

In this paper, we analyze BAT results in a group of patients with immediate allergic reactions to BLs using various DeAns as immunogens. We compare the results to those obtained using the free drug or hapten. We further analyze these results taking into account the structural features of the DeAns, using diffusion Nuclear Magnetic Resonance (NMR) and molecular dynamics simulation (MDS).

## 2. Results

In the penicillin allergy scenario, the formation of the antigenic determinant structures (or epitopes) is based on the nucleophilic attack by the amino groups of lysine residues in proteins on the electrophilic β-lactam ring. The antigenic determinants of benzylpenicillin and amoxicillin, BPO and AXO, respectively, have been previously characterized [17,21]. Their excellent stability enables their use for both clinical diagnostic purposes and in research. These antigenic determinants have been used to decorate dendrimers, generating BPO-DeAn and AXO-DeAn, respectively. Those conjugates were used to evaluate basophil activation in vitro in blood samples from confirmed allergic patients. 

### 2.1. Synthesis and Characterization of DeAn

**Figure 1:**
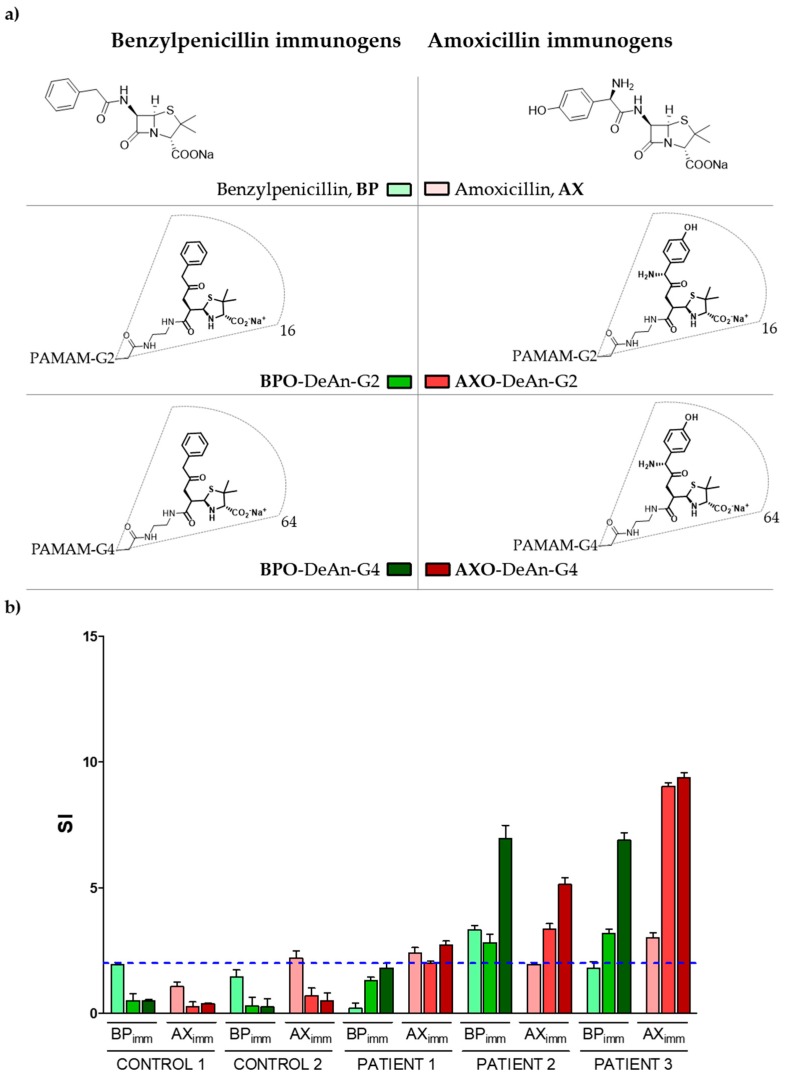
Immunogen structures and BAT results: (**a**) Chemical structure of the series of immunogens evaluated: the free drugs, BP and AX; DeAn-G2 decorated with BPO (BPO-DeAn-G2) or AXO (AXO-DeAn-G2) and DeAn-G4 decorated with BPO (BPO-DeAn-G4) or AXO (AXO-DeAn-G4); (**b**) Evaluation of BAT performed with different immunogens related to BP and AX: individual BAT results expressed as SI of each structure for patients and controls. BAT represents the mean of five determinations plus the mean + SEM (Standard Error of the Mean).

PAMAM dendrimers of different generations (G2 and G4) were peripherally functionalized with either BPO or AXO units, generating four DeAns, namely BPO-DeAn-G2, BPO-DeAn-G4, AXO-DeAn-G2 and AXO-DeAn-G4 (Figure 1a). All conjugates were fully characterized to confirm complete functionalization by means of ^1^H and ^13^C NMR [17,21]. 

The MDS study provided information on the size and shape of the DeAn conjugates. Both DeAn-G2 conjugates were found to be similar in size, as quantified by the radius of gyration (Rg), Table 1. The DeAn-G4 conjugates were also found to be similar sizes to each other. The shape of the macromolecules is indicated by the asphericities values (δ). Values can range between 1 (for a linear arrangement of atoms) and 0 (for shapes with high 3D similarity). The observed values (Table 1) imply that higher generation conjugates have more globular structures.

The conjugates were examined by diffusion NMR to estimate their size in aqueous solution. Diffusion coefficients (D) were determined with DOSY (difussion-ordered spectrocopy) eperiments and used to estimate size in Å by calculating the hydrodynamic radius (R_H_) using the Stokes-Einstein equation (Table 1) [29,30]. As expected, the larger DeAn-G4 conjugates diffused more slowly, resulting in higher R_H_ values than the smaller DeAn-G2 conjugates. 

The values calculated by MDS are in broad agreement with those obtained from the DOSY experiments, showing values of ~14 Å for DeAn-G2 and ~20–22 Å for DeAn-G4.

### 2.2. Clinical Evaluation

In order to study the potential of the different DeAns to be used as antigen in the BAT for the diagnosis of benzylpenicillin and amoxicillin allergy, we evaluated basophil activation through specific IgE recognition at the cell surface. This was achieved using BAT with blood samples from three amoxicillin-allergic patients and two individuals tolerant to this drug (Table 2), according to conventional diagnostic protocols applied in clinical practice (European Network of Drug Allergy, ENDA) [31]. 

BAT results (Figure 1b) were considered positive when the stimulation index (SI) was greater than two to at least one of the concentrations used. SI was calculated as the ratio between the percentage of activated basophils with the different immunogens (free drugs and DeAns), compared to the percentage activated with the negative control. Results for patient 1, who is allergic to AX but not BP, showed positive BAT results to the free drug (AX), as well as to the DeAn cojugates decorated with AXO units. Negative values were obtained with BP and both BP-derived DeAn conjugates. Results for patient 2, who is a cross-responder to BP and AX, showed a positive SI to only one free drug, BP, and all the DeAns. Similarly, data obtained for patient 3, a cross-responder to BP and AX, showed positivity to only one of the free drugs, in this case AX, and all the DeAn conjugates. In all cases, higher values of SI were obtained for DeAn-G4 than DeAn-G2. 

When analyzing the data for tolerant individuals (i.e., control subjects allergic to neither AX nor BP), a positive BAT result is observed in control 2 for the free molecule of AX, yielding a false positive result. However, it is important to note that negative test results were obtained using DeAns for both control individuals, even though they are allergic to other BLs (cephalosporins). Similarly, patient 1 only showed a positive BAT for the AX-derived molecules with no activation of any BP-derived conjugates. In terms of the chemical structure, additional amino and hydroxyl groups on the benzyl side chain of AX compared to the side chain of BP may explain the differential activation through IgE recognition at basophil surfaces. These preliminary results indicate a high specificity of the BAT when using DeAns as immunogens. The high specificity of sIgE recognition for BPO-DeAn and AXO-DeAn has already been demonstrated in immunoassays [17], however this is the first time they have been evaluated in cellular assays.

We evaluated whether the inclusion of these DeAns can improve the potential of BAT for diagnosing penicillin allergic patients. Employing the current available approach using the free drug alone, of the three patients allergic to AX (patients 1, 2 and 3) basophil activation occurred in two of them (patients 1 and 3), and of the two patients allergic to BP (patients 2 and 3) only patient 2 showed positive results. These ratios increase to three of three of patients using AXO-DeAn and two of two using BPO-DeAn, respectively. Thus, all patients were correctly diagnosed as allergic, demonstrating that the inclusion of DeAns improves test sensitivity. Moreover, sensitivity was also better than that obtained by RAST, which could only diagnose two of the three patients correctly (Table 2).

## 3. Discussion

The diagnosis of penicillin allergy is complex and there is a large and unmet need from health-care professionals for better in vitro methods. The sensitivity of the tests can be influenced by the structure of the immunogen and is related to the underlying mechanisms involved in the allergic process. Activation of effector cells, mast cells and basophils, requires antigens of a certain size [32] and may be negatively affected by the separation between the antigenic determinants [32,33]. In the case of drugs, they are thought to act as haptens, as they are considered too small to induce allergy by themselves. To reach the adequate size to induce reactions, penicillins must bind proteins covalently, forming conjugates [34,35,36]. The simultaneous recognition of a penicillin-protein conjugate by at least two sIgE molecules bound to adjacent FcεRI at the cellular surface is known as cross-linking. This induces degranulation of the effector cells, leading to the release of inflammatory mediators responsible for the reaction [35]. 

In the BAT scenario, the free drugs (AX or BP) are assumed to bind proteins present in blood covalently through β-lactam reactivity, forming a big enough conjugate to achieve cross-linking. This approach attempts to emulate in vivo conditions, however it lacks information about the chemical composition of the conjugate inducing the activation. By using DeAns, one has more control over conjugate size, multivalence and the structure of peripheral antigenic determinants, allowing more reproducible assays. The use of the appropriate DeAn structure ensure the optimal interaction between the drug moieties and sIgE on the basophil surface, inducing more potent basophil degranulation and improving BAT sensitivity. 

These relationships between cell activation and immunogen structure have been recently explored in in vitro studies performed in animal models using 2,4-dinitrophenyl (DNP) as a model hapten. It was found that the number of epitopes and the distance between them in synthetic nanostructures have different effects on mast cell degranulation [32,37,38,39]. One of these studies, using dendrimers decorated with DNP epitopes, showed that larger DNP_16_-dendrimers (64 Å) trigger mast cell degranulation by cross-linking IgE-receptor complexes, whereas smaller DNP-dendrimers are inhibitory [39]. Other studies have analyzed these relationships in BLs using monovalent haptens, which could be recognized by IgE but unable to bind two adjacent antibodies simultaneously. These structures were shown to inhibit the development of an allergic reaction [31,40,41], for both in vitro and in vivo tests in BP allergic patients, however this finding has not been further explored in a clinical setting. Most of our current knowledge regarding the activation of nanostructures in effector cell degranulation is based on studies performed with model ligands and animal mast cells. To the best of our knowledge, no studies employing this strategy have been used with real haptens or in human samples.

These studies have provided much needed information about the requirements necessary to activate effector cells, which cannot be deduced using the free drug alone. In fact, little is known about the nature of the adduct that activates basophils in the assay. In this context, the size of the actual carrier protein, the number of reactive sites, and the proximity between them are considered key factors that influence the cross-linking process, and their evaluation would be very complex. The use of well-defined nanostructures with consistent sizes and epitope density will provide a valuable tool to study the structural parameters required for these cell processes. We evaluated the ability of these nanostructures to stimulate basophils and we found that DeAns were able to induce activation in a selective and specific way. Interestingly, basophils from allergic patients follow similar patterns regarding generation: DeAn-G4 produces higher SI than DeAn-G2; this could be due to the size, valence or proximity between epitopes in the immunogen: compared to DeAn-G2, DeAn-G4 have a higher size (~20 Å vs. ~14 Å), an increased density of epitopes (64 vs. 16) and a higher proximity between them, favoring the IgE cross-linking on cell surface, and enhancing activation. This preliminary data suggests that using a size of approximately 20 Å as well as a higher density of epitopes may better resemble the IgE molecular recognition that occurs with penicillin-protein conjugates formed in vivo.

## 4. Materials and Methods 

### 4.1. Molecular Dynamic Simulation (MDS)

*Dendrimer Building*. AXO-DeAn-G2 was produced using the previously described procedure [17]. 

*Simulation Details*. Full atomistic simulation was performed in water as explicit solvent at neutral pH. We used the AMBER 12 MD software package for all calculations [42]. To preserve overall charge neutrality and represent more realistic conditions, an appropriate number of Na^+^ and Cl^−^ counterions were added and the molecules hydrated, using the TIP3P water model [43], in a truncated octahedral cell. The dimensions of the cell were chosen to provide a minimum 10 Å solvation shell around the dendrimer structure. 

Solvated structures were minimized as described previously [44], using six cycles of conjugated gradient minimization. During the initial cycle, dendrimers were kept in their starting conformation using a harmonic constraint with a force constant of 500 kcal/mol-Å^2^. This was followed by another five periods of minimization while decreasing the harmonic restraint force constant from 20 kcal/mol-Å^2^ to zero in steps of 5 kcal/mol-Å^2^. To allow a slow relaxation of the assembled dendrimer-solvent system, the minimized structure was heated slowly from 0 to 300 K with three steps of 40 ps of MD, the first of them under conditions of constant volume-constant temperature (NVT) and the rest under constant pressure-constant temperature (NPT) conditions. Initially, we applied a weak 20 kcal/mol-Å^2^ harmonic constraint to the solute starting structure and slowly decreased it to zero in 5 kcal/mol-Å^2^ steps. At this point the dendrimer relaxation was determined from the autocorrelation function of the squared radius-of-gyration.

Finally, we carried out 2 ns of unconstrained MD simulation in NPT ensemble to equilibrate the system at 300 K. To solve the motion equation we used the Verlet leapfrog algorithm [44], with an integration step of 2 fs. Bond lengths involving bonds to hydrogen atoms were constrained using the SHAKE algorithm [45], using a geometrical tolerance of 5 × 10^−4^ Å.

Finally, starting from the configurations generated by the above procedure, production runs of 20 ns trajectories were performed under an NPT ensemble. Temperature regulation was achieved using the Berendsen weak coupling method (1 ps time constant for heat bath coupling and 0.5 ps for pressure relaxation time) [46]. The particle-mesh Ewald (PME) algorithm was employed to treat long-range electrostatic interactions [47], with a real space cut off of 9 Å. The same cutoff was used for van der Waals interactions. For the structural analyses (*Rg*, *dendrimer shape*, etc.), the last 1 ns equilibrated trajectory was used. Amber modules ptraj and cpptraj were used to accomplish these analyses. VMD software was used for the calculation of molecular surfaces [48]. 

### 4.2. DOSY Nuclear Magnetic Resonance (NMR) Experiments

The samples were prepared in deuterium oxide at a concentration of.0.5 mM (within the infinite dilution range for similar samples at 0.1–2.1 mM) [30]. The experiments have been performed on a The Bruker Ascend^TM^ 400 MHz spectrometer, equipped with a 5 mm BBFO^PLUS^ probe with ^2^H “lock” channel and z gradient. The spectrometer is also equipped with a control temperature unit prepared to work at temperatures ranging from 0 °C to +50 °C. Gradient strength was calibrated by measuring the diffusion rate of pure water of residual protons in D_2_O. All experiments were conducted at 300 K. The samples were allowed to equilibrate for no fewer than 15 min.

To determine the diffusion rates, a 2D sequence using double stimulated echo for convection compensation and LED using bipolar gradient pulses for diffusion was used.

The diffusion coefficients determined were used to calculate the hydrodynamic radius via the Stokes-Einstein equation: $R_{H}=K_{B}T/6\pi \eta D$, were *K_B_* is the Boltzmann constant, *T* is the temperature and *η* is the viscosity of the solution (1.0963 cP for D_2_O viscosity) [30].

### 4.3. Patients

Subjects, 2 tolerant and 3 with an immediate allergic reaction to penicillins, were included in the study. Diagnosis was confirmed following the European Academy of Allergy and Clinical Immunology (EAACI)/European Network for Drug Allergy (ENDA) guidelines [49]. Patients were classified into two groups: cross-reactors when they recognized AX as well as major and minor determinants of BP by skin testing, RAST or drug provocation test; and selective reactors when positive to AX, although negative to determinants of BP.

The study was conducted according to the Declaration of Helsinki principles and was approved by the Provincial Ethics Committee of Malaga. All subjects included in the study were informed orally and signed the corresponding informed consent.

### 4.4. Basophil Activation Tests

**Figure 2:**
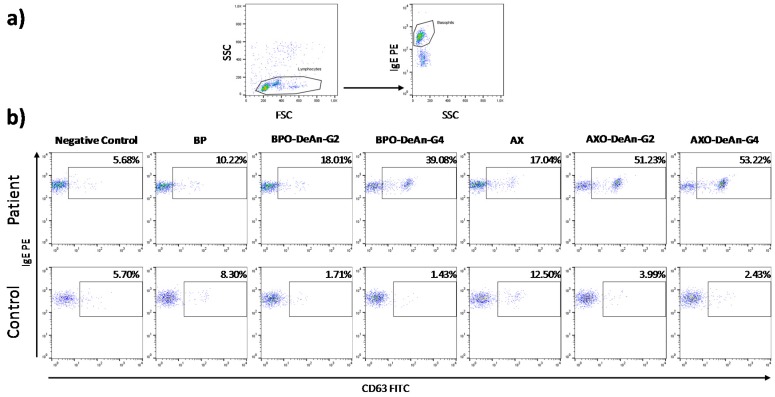
Cytometry dot plots showing: (**a**) basophils selection strategy; (**b**) a representative example (patient 3 and control 2) of results for all the immunogens tested, including native drug and DeAns.

Basotest (Orpegen Pharma, Heidelberg, Germany) was used following the manufacturer’s recommendations and previous procedures [26]. Briefly, 100 µL of heparinized whole blood was incubated with stimulation buffer for 10 min at 37 °C in a water bath. After this, 100 µL of the washing solution was added to the negative control tube and fMLP (chemotactic peptide *N*-Formyl-Met-Leu-Phe) to the positive control tube. Samples were incubated with two immunogen concentrations: BP at 2 and 0.5 mg/mL; AX at 1.2 and 0.25 mg/mL and DeAns at 1 and 0.1 mg/mL (chosen on the basis of previous dose–response curves and cytotoxicity studies). The samples were incubated for 20 min at 37 °C in a water bath. The degranulation was stopped by incubating the samples on ice for 5 min and then 20 µL of staining reagent containing two monoclonal antibodies, anti-IgE PE and antigp53 FITC (gp53 is a glycoprotein expressed on activated basophils), was added to each tube and incubated for 20 min in an ice bath covered to prevent exposure to light. The cells were analyzed in a flow cytometer by acquiring at least 1000 basophils per sample. Results were considered as positive when the SI, calculated as the ratio between the percentage of degranulated basophils with the different haptens and the negative control, was greater than two to at least one of the dilutions mentioned above. When the percentage of spontaneously activated basophils was less than 2.5%, the percentage of basophils activated after contact with the antigen should be equal to or greater than 5%. The BAT selection strategy of representative samples for a patient and a control is shown in Figure 2.

## 5. Conclusions

The study of the structural requirements of conjugates for basophil activation can define the suitable molecules needed to perform BAT with increased sensitivity. Structural properties of DeAn have shown to be appropriate for intra-molecular cross-linking of sIgE bound to FcεRI on basophils. This preliminary study has allowed, for the first time, the performance of BAT with molecules other than the free drug, which enhances the response by way of a better control of the method in terms of reproducibility regarding the immunogen size and density of epitopes. Nothing similar designed at the nanoscale focused on basophils has been undertaken, and potential application to diagnosis are foreseeing, in terms of sensitivity of the test.

## Figures and Tables

**Table 1:** Radius of gyration *Rg*, aspect ratios (*Iz*/*Ix* and *Iz*/*Iy*), asphericities (*δ*) [calculated by MDS] [17,21], diffusion coefficients (*D*) and hydrodynamic radius (*R_H_*) [determined by NMR experiments] of DeAns.

Dendrimer	MDS				NMR Experiments	
	*Rg* (Å)	*Iz*/*Ix*	*Iz*/*Iy*	*δ*	*D* (m^2^s^−1^)	*R_H_* (Å)
BPO-DeAn-G2	14.20	2.40	2.40	0.054	1.38 × 10^−10^	14.50
BPO-DeAn-G4	21.90	1.25	1.17	0.005	1.00 × 10^−10^	20.04
AXO-DeAn-G2	13.65	1.74	1.20	0.024	1.40 × 10^−10^	14.32
AXO-DeAn-G4	22.03	1.61	1.07	0.019	1.00 × 10^−10^	20.04

**Table 2:** Classification and clinical characteristics of controls and patients diagnosed with an immediate allergic reaction to AX included in the study.

Subject	Sex	Age (Years)	Reaction	Responsible Drug	Int R-S	Skin Test				RAST	
						BP-OL	MD	AX	+to Other Drug	BPO-PLL	AXO-PLL
Contr 1	F	43	Urticaria/AE	Cefaclor	5	−	−	−	Cefaclor	−	−
Contr 2	F	48	Anaphylaxis	Cefur	3	−	−	−	Cefur	−	−
Pat 1	M	28	Anaphylaxis	AX	5	−	−	+	nd	−	−
Pat 2	M	30	Anaphylaxis	AX	5	nd	nd	nd	nd	+	+
Pat 3	F	18	Anaphylaxis	BP	6	+	-	+	nd	+	+

Abbreviations. Contr: Control; Pat: Patient; F: female; M: male; AE: Angioedema; AX: Amoxicillin; BP: Benzylpenicillin; Cefur: Cefuroxime; Int R-S: Time interval between reaction and in vitro study (months); BP-OL: benzylpenicilloyl-octa-l-lysine; MD: benzylpenicillin minor determinant; (−): negative; (+): positive; nd: not determined; RAST: Radioallergosorbent test; BPO-PLL: benzylpenicilloyl-poly-l-lysine; AXO-PLL: amoxicilloyl-poly-l-lysine.
